# Applications and Comparisons of Four Time Series Models in Epidemiological Surveillance Data

**DOI:** 10.1371/journal.pone.0088075

**Published:** 2014-02-05

**Authors:** Xingyu Zhang, Tao Zhang, Alistair A. Young, Xiaosong Li

**Affiliations:** 1 Department of Medical Statistis, West China School of Public Health, Sichuan University, Chengdu, Sichuan, P.R. China; 2 Department of Anatomy with Radiology, University of Auckland, Auckland, New Zealand; University of Florida, United States of America

## Abstract

Public health surveillance systems provide valuable data for reliable predication of future epidemic events. This paper describes a study that used nine types of infectious disease data collected through a national public health surveillance system in mainland China to evaluate and compare the performances of four time series methods, namely, two decomposition methods (regression and exponential smoothing), autoregressive integrated moving average (ARIMA) and support vector machine (SVM). The data obtained from 2005 to 2011 and in 2012 were used as modeling and forecasting samples, respectively. The performances were evaluated based on three metrics: mean absolute error (MAE), mean absolute percentage error (MAPE), and mean square error (MSE). The accuracy of the statistical models in forecasting future epidemic disease proved their effectiveness in epidemiological surveillance. Although the comparisons found that no single method is completely superior to the others, the present study indeed highlighted that the SVMs outperforms the ARIMA model and decomposition methods in most cases.

## Introduction

Public health surveillance is an important way to continuously collect, analyze, interpret and disseminate health data essential to prevention and control [1]. Public health surveillance systems are designed to facilitate the detection of abnormal behavior of infectious diseases and other adverse health events. To achieve this goal, different statistical methods have been used to forecast infectious disease incidence. Time series models have long been of interest in the literature. The time series models try to predict epidemiological behaviors by modeling historical surveillance data. Many researchers have applied different time series models to forecasting epidemic incidence in previous studies. Exponential smoothing [2] and generalized regression [3] methods were used to forecast in-hospital infection and incidence of cryptosporidiosis respectively. Decomposition methods [4] and multilevel time series models [5] were used to forecast respiratory syncytial virus. Autoregressive integrated moving average (ARIMA) models have been widely used for epidemic time series forecasting including the hemorrhagic fever with renal syndrome [6], [7], dengue fever [8], [9], and tuberculosis [10]. Models based on artificial neural networks were also used to predict the incidence of hepatitis A [11], [12] and typhoid fever [13].

The decomposition methods are generally the most traditional methods in time series analysis [14], [15]. These methods try to break down the original series into a long trend pattern, a seasonal pattern and residuals. Seasonal indices are extracted to express the seasonal pattern; a regression model is established to express the long trend pattern and the residuals are ignored in the methods. Because the decomposition time series methods do not involve a lot of mathematics or statistics, they are relatively easy to explain to the end user. This is a major advantage because if the end user has recognition of how the forecast was developed, he or she may have more confidence in its use for decision making.

The ARIMA models are almost the most widely used methods [16], [17]. The ARIMA models are generally derived from three basic time series models (1) autoregressive (AR), (2) moving average (MA), and (3) autoregressive moving average (ARMA). The current value of the time series is a linear function of its previous values and random noise in the AR model; whereas the current value of the time series is a linear function of its current and previous values of residuals in the MA model. The ARMA model is the combination of AR and MA, which considers both the historical values and residuals. The time series required in AR, MA, and ARMA models are stationary processes. This means that the mean and the covariance of the series do not change with time. Transformation of the series into a stationary one has to be performed first for non-stationary time series. The ARIMA model fits the time series data generally based on the ARMA model and a differencing process which effectively transforms the non-stationary data into a stationary one.

In recent years, machine learning based time series models such as artificial neural networks have been successfully applied for modeling infectious disease incidence time series [18]. Support vector machines (SVMs) are a new type of machine learning methods based on statistical learning theory [19]. They could lead to greater potential and better performance in practical applications. This is due to the structural risk minimization principle employed in SVMs, which has greater generalization ability and is superior to the empirical risk minimization principle that is adopted by traditional neural networks. SVMs have been successfully applied in different problems of time series prediction such as forecasting production value in machinery industry [20], predicating engine reliability [21] and economic time series predication [22], [23]. The successful utilization of support vector machines in time series predication motivates our research work by using support vector machines for epidemic time series forecasting.

The objectives of the present paper are to compare four typical time series methods, namely, two decomposition methods (regression and exponential smoothing), ARIMA model and SVMs in theory and practice as well as their real forecasting efficacy in epidemic time series. This comparison may be helpful for the epidemiologist to choose the most suitable methodology in a given situation.

## Materials and Methods

### Materials

We gathered available monthly incidence of nine typical infectious diseases time series data which were reported by the Chinese Center for Disease Prevention and Control (CDC). The data were collected from the Chinese National Surveillance System established in 2004. The incidence time series of brucellosis, gonorrhea, hemorrhagic fever renal syndrome (HFRS), hepatitis A (HA), hepatitis B (HB), scarlet fever, schistosomiasis, syphilis, typhoid fever from 2005 to 2012 were collected.

### Methods

#### Decomposition methods

The decomposition methods try to extract the underlying pattern in the data series from randomness. The underlying pattern then can be employed to predict future trends and make forecasts. The underlying pattern can also be broken down into sub patterns to identify the component factors that influence each of the values in a series. Two separate components of the basic underlying pattern that tend to characterize the infectious disease time series are usually identified in decomposition methods. They are the trend cycle and seasonal factors. The trend cycle represents long term changes, and the seasonal factor is the periodic fluctuations with constant length that is usually caused by known factors such as rainfall, month of the year, temperature, timing of the holidays, etc. The decomposition model assumes that the data has the following form:

Time series  =  Pattern + Error  =  Trend cycle+ Seasonality+ error

The seasonality part of the time series is usually expressed with the seasonal indices [24]. To arrive at seasonal factors, the entire incidences for the training sample are averaged first, and then the averaged incidence is divided by the mean incidence for each month. If the seasonal index is bigger than 1, it means that the incidence is usually higher than the average level. Otherwise, it means that the incidence is usually lower than the average level.

Once the Seasonal indices are calculated, one can deseasonalize data by dividing by the corresponding index.

Deseasonalized data  =  Raw data/Seasonal Index

The long-term trend is estimated from the deseasonalized data. There are many ways to estimate the long-term trend, such as moving average, exponential smoothing, and linear regression. In simple moving average methods, the current value is calculated as the mean of its previous *k* values, whereas exponential smoothing assigns exponentially decreasing weights over time. When the time series *x*(*t*) begins at time *t* = 0, the simplest form of exponential smoothing is given by the formulae: 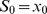


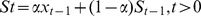
where 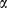 is the smoothing factor and 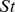 is the output of the exponential smoothing algorithm 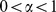.

The linear regression method is another simple way to express the long term trend in which a common linear regression model is established between the incidence and time *t*.

#### ARIMA model

The ARIMA model originated from AR model, MA model, and the combination of AR and MA, the ARMA models [25]. AR models express the current value of the time series *X*(*t*) linearly in terms of its previous values (*X*(*t*−1), *X*(*t*−2)…) and the current residuals 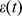, which can be expressed as: 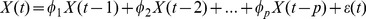
(1)


MA models express the current value of the time series *X*(*t*) linearly in terms of its current and previous residual series 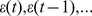. the model can be expressed as: 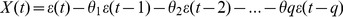
(2)


ARMA models are a combination of AR and MA models, in which the current value of the time series is expressed linearly in terms of its previous values as well as current and previous residual series. It can be expressed as: 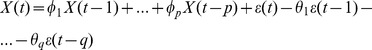
(3)


The ARIMA model deals with non-stationary time series with differencing process based on the ARMA model. The differenced stationary time series can be modeled as ARMA model to yield ARIMA model.

The ARIMA model is usually termed as ARIMA (*p, d, q*)×(*P, D, Q*)*_S_*. In the expression, *P* is the seasonal order of autoregressive, *p* the non-seasonal order of autoregressive, *Q* the seasonal order moving average, *q* the non-seasonal order of moving average, *d* the order of regular differencing and *D* the order of seasonal differencing. The subscripted letter “*s*” indicates the length of seasonal period. For example, the incidence of infectious disease varies in the annual cycle, so *s* = 12 in the present study.

The ARIMA modeling procedure consists of three iterative steps: identification, estimation, and diagnostic checking. Prior to fitting the ARIMA model, an appropriate difference of the series is usually performed to make the series stationary. Identification is the process of determining seasonal and non-seasonal orders using the autocorrelation functions (ACF) and partial autocorrelation functions (PACF) of the transformed data [26]. The ACF is a statistical tool that measures whether earlier values in the series have some relation to later values. PACF captures the amount of correlation between a variable and a lag of the said variable that is not explained by correlation at all low-order lags. Parameters in the ARIMA model(s) are estimated with the conditional least squares (CLS) method [27] after the identification step. Finally, the adequacy of the established model for the series is verified by employing white noise tests [28] to check whether the residuals are independent and normally distributed. It is possible that several ARIMA models may be identified, and the selection of an optimum model is necessary. Such selection of models is usually based on the Akaike Information Criterion (AIC) and Schwartz Bayesian Criterion (SBC) [29].

#### Support Vector Machine

SVMs estimate the regression using a set of linear functions that are defined in a high dimensional space. SVMs carry out the regression estimation by using Vapnik's 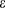-insensitive loss function. SVMs use a risk function consisting of the empirical error and a regularization principle [30].

Assume that 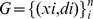 is a set of data points, where 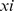 is the input sample, 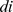 is the desired value and *n* is the total number of data. The SVMs calculate the function using the following: 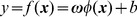
(4)where 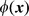 is the high dimensional feature space which is non-linearly mapped from the input space 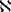. The coefficients 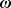 and *b* are calculated by minimizing 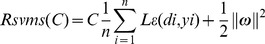
(5)

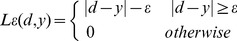
(6)


In eq. (5), the first term 
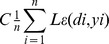
 represents the empirical error risk, which is calculated by the 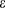-insensitive loss function in eq. (6). The second term 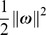 is the regularization term. *C* is the regularized constant, which determines the trade-off between the empirical risk and the regularization term. If the value of *C* is changed, the relative importance of the empirical risk and the regularization term will also be changed. Increasing the value of *C* will lead to the growth of the weight of the regularization. 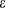 is named as the tube size, which is equivalent to the approximation accuracy placed on the training data sample. Both C and 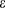 are user-prescribed parameters [31].

To estimate 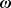 and *b*, eq. (5) is transformed to the primal function given by eq. (7) by introducing the positive slack variables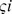 and 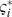 as follows:
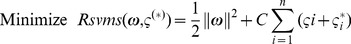
(7)


Subjected to 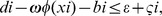


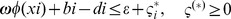



Finally, by introducing Lagrange multipliers and exploiting the optimality constraints, the decision function given by Eq. (4) has the following explicit form:
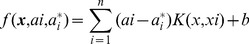
(8)


In Eq. 5, 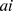 and 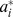 are the so-called Lagrange multipliers. They satisfy the equalities 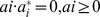, and 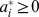 where *i* = 1,…,*n*, and are obtained by maximizing the dual function of eq.4 which has the following form: 
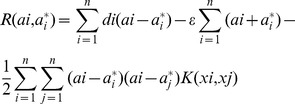
(9)with the constraints 
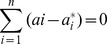


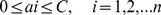


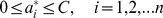




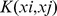
 is called the kernel function. The value of the kernel is equal to the inner product of two vectors 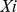 and 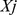 in the feature space 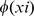 and 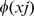, that is 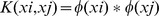. The elegance of using the kernel function is that one can deal with feature spaces of arbitrary dimensionality without having to compute the map 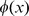 explicitly. A. Typical examples of kernel function are the Gaussian kernel 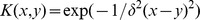 where 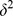 is the bandwidth of the Gaussian kernel [32]. The kernel parameter should be carefully chosen as it implicitly defines the structure of the high dimensional feature space 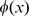 and thus controls the complexity of the final solution. From the implementation point of view, training SVMs is equivalent to solving a linearly constrained quadratic programming (QP) with the number of variables twice as that of the training data points. The sequential minima optimization algorithm propounded by Scholkopf and Smola [33], [34] is reported to be very effective in training SVMs for solving regression problems.

#### Model selection criterion and evaluation indices

The contrasts between the observed value of the raw series and the predicted values obtained through the four methods were compared to determine the efficacy of the four forecasting methods used in the present study. The mean absolute error (MAE), mean absolute percentage error (MAPE), and the root mean square error (RMSE) were selected as the measures of evaluation because as empirical methods they are widely used in combining and selecting forecasts for measuring bias and accuracy of models [35].

These measures were calculated using Equations (10), (11), and (12). *Pt* is the predicted value at time *t*, *Zt* is the observed value at time *t* and *T* is the number of predictions. 
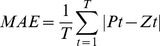
(10)

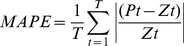
(11)

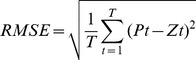
(12)


To take into account the variability of MAE, MAPE and RMSE, the block bootstrap technique [36] was adopted to calculate their standard errors. All of the incidence time series in the current research have a one-year period of seasonality (*D* = 1). Therefore, in our block bootstrap simulations, the block length was set to be 12 months so that the autocorrelation structure within seasonal blocks was reserved. We firstly simulated 10000 replications by block bootstrap sampling, and then calculated the MAE, MAPE and RMSE for each replication. At last, the standard errors could be obtained by the following formula: 
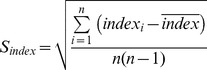



where *n* is number of replications (10000), *index* could be MAE, MAPE or RMSE. Take MAE as an example, here *Index* means MAE, 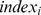 represents the specific value of MAE in the *i*-th replication and 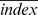 is the mean value of MAE for the whole replications. It is the same with MAPE and RMSE.

## Time Series Modeling Results

### Decomposition Methods

**Figure 1:**
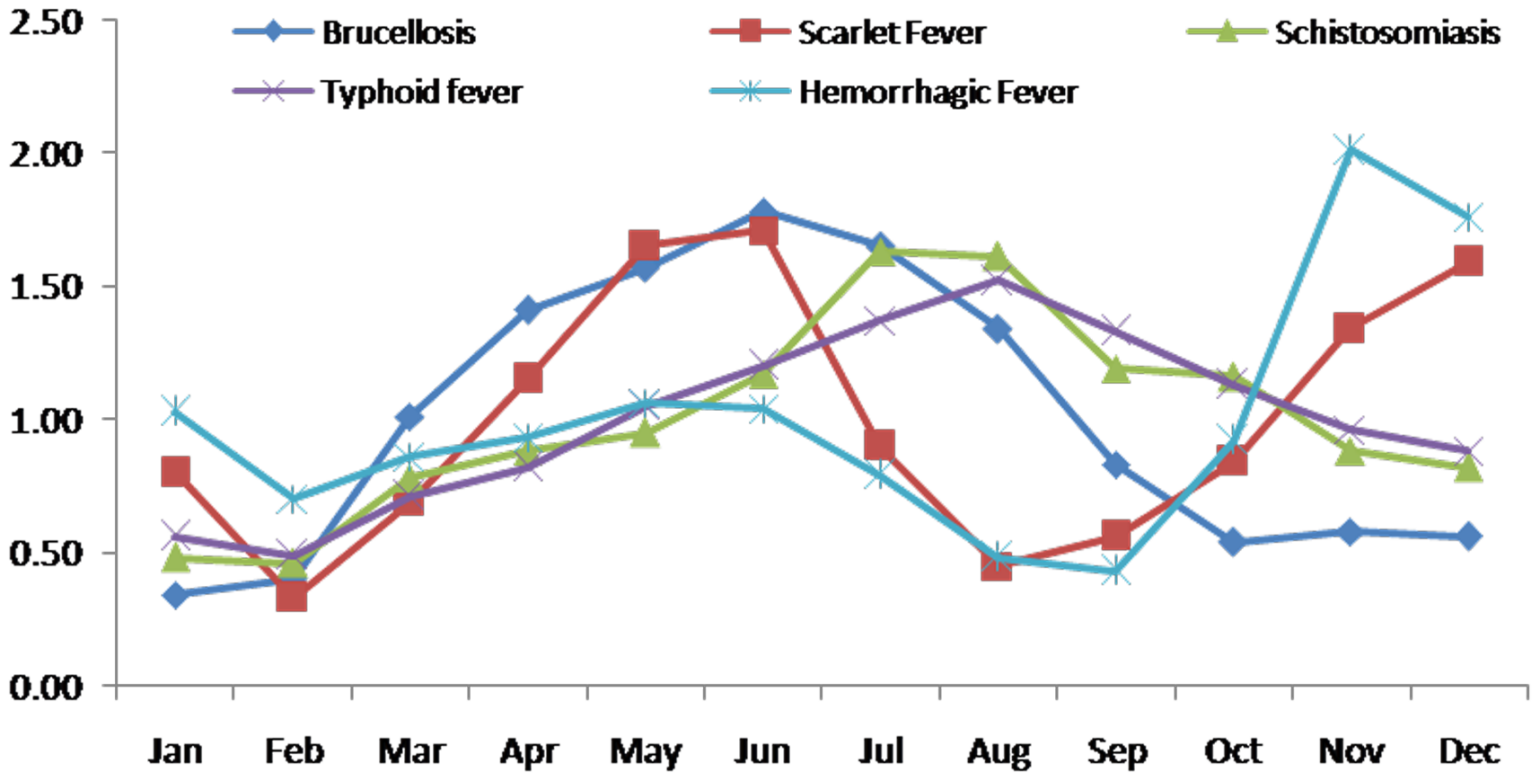
Seasonal index of each type of infectious disease (1).

**Figure 2:**
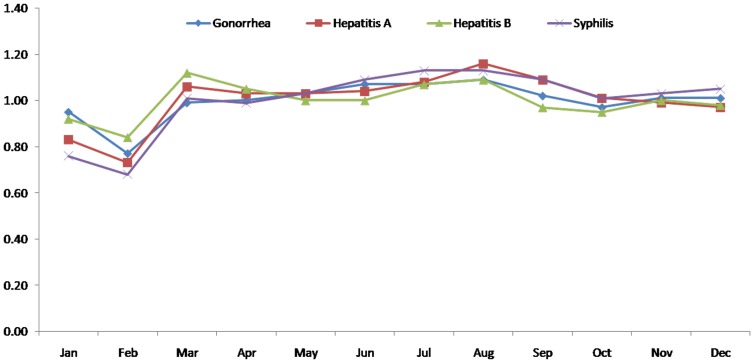
Seasonal index of each type of infectious disease (2).

Seasonal indices of different types of infectious diseases were extracted from the original time series, which are listed in Table 1 (Seasonal index of each type of infectious disease), Figure 1–2(Seasonal index of each type of infectious disease (1)). The seasonality of the incidence behavior of each infectious disease can be seen according to the seasonal indices. All the infectious diseases selected show a seasonal trend as the occurrence of infectious disease can be more or less influenced by the temperature, rainfall and sunshine, etc. However, the extent of the seasonality is not quite similar among them. Figure 1 shows the five types of diseases whose seasonal index varies obviously through 12 months. Figure 2 shows the four types of disease whose seasonality indices do not vary seriously. Brucellosis, hemorrhagic fever, scarlet fever, schistosomiasis and typhoid fever show stronger seasonality than the others, as their variances of their seasonal indices are bigger than others. The incidence of brucellosis is higher in summer and lower in winter, with the crest in June. Hemorrhagic fever has the highest seasonal index in November and lowest in September. Scarlet fever has the higher seasonal index in May, June and December and lower index in August. The incidence of schistosomiasis is higher in summer and lower in winter, with the crest in July. The incidences of typhoid fever are higher in summer and lower in winter, with the crest in August. The other diseases, such as Hepatitis A, Hepatitis B, Gonorrhea and syphilis have relatively smooth seasonal index curves.

**Table 1:** Seasonal index of each type of infectious disease.

	Jan	Feb	Mar	Apr	May	Jun	Jul	Aug	Sep	Oct	Nov	Dec
Brucellosis	0.34	0.40	1.01	1.41	1.57	1.78	1.65	1.34	0.83	0.54	0.58	0.56
Gonorrhea	0.95	0.77	0.99	1.00	1.03	1.07	1.07	1.09	1.02	0.97	1.01	1.01
Hemorrhagic Fever	1.03	0.70	0.86	0.93	1.06	1.04	0.79	0.48	0.43	0.92	2.01	1.76
Hepatitis A	0.83	0.73	1.06	1.03	1.03	1.04	1.08	1.16	1.09	1.01	0.99	0.97
Hepatitis B	0.92	0.84	1.12	1.05	1.00	1.00	1.07	1.09	0.97	0.95	1.00	0.98
Scarlet Fever	0.80	0.33	0.69	1.15	1.65	1.71	0.90	0.45	0.56	0.84	1.34	1.59
Schistosomiasis	0.48	0.46	0.78	0.88	0.95	1.17	1.63	1.61	1.19	1.16	0.88	0.82
Syphilis	0.76	0.68	1.01	0.99	1.03	1.09	1.13	1.13	1.09	1.01	1.03	1.05
Typhoid fever	0.56	0.49	0.71	0.82	1.05	1.20	1.37	1.52	1.33	1.13	0.96	0.88

After the extraction of seasonal indices, linear regressions were modeled for the rest of the incidence time series. The form of the regression model is:

Deseasonalized value at time *t* =  Constant + Coefficient * *t*

The parameters of the established models are listed in Table 2 (Regression results of each series removed seasonality). *R^2^* is the coefficient of determination. It ranged between 0 and 1, which is used to describe how well a regression line fits a set of data. An *R^2^* near 1 indicates that a regression line fits the data well, while an *R^2^* closer to 0 indicates a regression line does not fit the data very well. It can be seen from Table 2 that the regression model on the seasonality-removed incidence data of brucellosis, gonorrhea, hepatitis A, Syphilis and typhoid fever generally fit well. The regression model on the seasonality removed incidence data of hepatitis B fit badly, and *P* value is over 0.05, however, the model is still used to forecast the incidence in the study as the model has good fitting and forecasting efficacy.

**Table 2:** Regression results of each series removed seasonality.

	Constant	Coefficient	R^2^	*p*
Brucellosis	0.0931	0.0022	0.7404	0.0011
Gonorrhea	1.1952	−0.0077	0.9220	0.0030
Hemorrhagic Fever	0.1218	−0.0010	0.5295	0.0005
Hepatitis A	0.5496	−0.0044	0.7991	0.0030
Hepatitis B	7.9058	0.0012	0.0019	0.4313
Scarlet Fever	0.1457	0.0013	0.1340	0.0067
Schistosomiasis	0.0193	0.0001	0.1975	0.0001
Syphilis	0.6958	0.0242	0.9464	0.0201
Typhoid fever	0.2121	−0.0019	0.8049	0.0005

We also used exponential smoothing to extract the long term trend after the extraction of seasonal indices. Different smoothing factors were tested from 0.1 to 0.9 with 0.1 step. Smoothing factors were selected by the criterion of minimum MSE in the modeling process.

### ARIMA model

ARIMA models were fitted to the nine types of infectious diseases from 2005 to 2011 and tested by predicting the incidence for the year 2012. Different ARIMA models were tested to determine the best fitting models. Table 3(Estimation of available ARIMA models for each disease) presents the results of the estimations using various ARIMA processes for the nine diseases incidence time series. The selections of the best models were performed according to the principle of AIC and SBC. The final selected ARIMA model was marked into yellow in Table 3. The parameter significance test and the white noise diagnostic check for residuals obtained by the selected model were made to ensure that the data was fully modeled.

**Table 3:** Estimation of available ARIMA models for each disease.

Disease	Identification	AIC	SBC
Brucellosis	ARIMA(0,0,0)×(0,1,1)	−282.39	−280.13
	***ARIMA(0,0,0)×(1,1,0)***	***−279.25***	***−276.99***
Gonorrhea	ARIMA(0,0,1)×(0,1,0)	−152.05	−149.79
	ARIMA(0,0,1)×(0,1,0)	−165.46	−163.08
	ARIMA(1,0,0)×(0,1,1)	−160.70	−156.18
	***ARIMA(0,0,1)×(1,1,0)***	***−168.48***	***−163.96***
Hemorrhagic Fever	ARIMA(1,0,0)×(0,1,0)	−354.63	−352.32
	ARIMA(0,0,1)×(0,1,0)	−358.07	−355.80
	***ARIMA(1,0,1)×(0,1,0)***	***−361.20***	***−356.67***
Hepatitis A	ARIMA(1,0,1)×(0,1,0)	−227.38	−222.85
	ARIMA(0,0,0)×(1,1,0)	−237.12	−234.85
	ARIMA(1,0,1)×(0,1,1)	−241.85	−235.06
	***ARIMA(0,0,0)×(0,1,1)***	***−241.90***	***−239.63***
Hepatitis B	ARIMA(1,0,0)×(0,1,0)	168.09	170.35
	ARIMA(0,0,1)×(0,1,0)	160.53	162.79
	ARIMA(1,0,1)×(0,1,0)	157.30	161.82
	ARIMA(2,0,0)×(0,1,0)	161.89	166.41
	ARIMA(3,0,0)×(0,1,0)	157.38	164.17
	ARIMA(0,0,2)×(0,1,0)	155.18	159.70
	ARIMA(1,0,0)×(1,1,0)	151.74	156.27
	***ARIMA(0,0,1)×(0,1,1)***	***134.61***	***139.13***
Scarlet Fever	ARIMA(1,0,0)×(0,1,0)	−169.68	−167.41
	ARIMA(0,0,1)×(0,1,0)	−172.22	−169.96
	ARIMA(0,0,1)×(0,1,1)	−190.10	−185.57
	ARIMA(1,0,0)×(1,1,0)	−173.30	−168.77
	ARIMA(0,0,1)×(1,1,0)	−176.39	−171.87
	ARIMA(2,0,0)×(0,1,0)	−179.34	−174.81
	***ARIMA(1,0,0)×(0,1,1)***	***−187.63***	***−183.15***
Schistosomiasis	ARIMA(1,0,0)×(0,1,0)	−517.69	−515.43
	ARIMA(1,0,1)×(0,1,0)	−524.15	−519.62
	ARIMA(1,0,0)×(0,1,1)	−520.67	−516.15
	***ARIMA(0,0,1)×(0,1,0)***	***−522.71***	***−520.45***
Syphilis	ARIMA(1,0,0)×(0,1,0)	−55.74	−53.48
	ARIMA(0,0,1)×(0,1,0)	−67.99	−65.74
	ARIMA(1,0,1)×(0,1,0)	−72.93	−68.41
	ARIMA(1,0,0)×(0,1,1)	−74.20	−69.67
	ARIMA(0,0,1)×(1,1,0)	−76.85	−72.33
	ARIMA(1,0,1)×(1,1,0)	−81.47	−74.69
	ARIMA(2,0,0)×(0,1,0)	−60.71	−56.19
	ARIMA(3,0,0)×(0,1,0)	−68.56	−61.77
	ARIMA(0,0,2)×(0,1,0)	−72.07	−67.54
	ARIMA(2,0,0)×(1,1,0)	−74.93	−68.14
	ARIMA(2,0,0)×(0,1,1)	−79.18	−72.39
	***ARIMA(1,0,1)×(0,1,1)***	***−83.70***	***−76.91***
Typhoid fever	ARIMA(0,0,1)×(0,1,0)	−369.44	−367.17
	***ARIMA(1,0,0)×(0,1,0)***	***−370.33***	***−368.07***

(**Note**: The final selected ARIMA model was marked into bold and italics).

### Support Vector Machine

The training number of the SVM based time series model needed to be determined. In previous studies [13], the training number for the training of periodic series is usually the period of the series. In the present study, the period of the entire infectious disease incidence selected is twelve. Therefore, twelve was selected as the training number for SVM based models, in which the last 12 months of data were reserved as the input for forecasting the present data. Proper transition of the data series is always necessary to determine the input and the output data before the training process. Supposing that *X_t_* represents the value at time *t*, the input matrix and the corresponding output matrix of the training and validation sample used in our study are written as follows: 
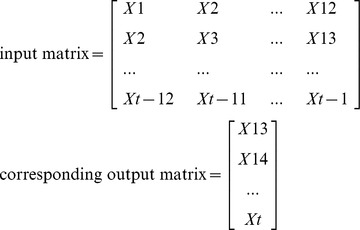



The input matrix is sent into SVM for training, and its corresponding output matrix is its training goal. Once the parameters are determined, they are used to forecast the incidence in 2012 iteratively.

Several parameters needed to be determined. They are *C*, 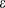 and the kernel parameter 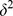. The value of 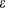 is reportedly not sensitive to the accuracy of SVMs. In the present study, the value of 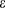 was prescribed as 0.01. Different *C and* 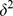 were examined from 2^−10^ to 2^10^ in 2 increments. There is no structural way to determine the optimal parameters of SVMs. In the present study, cross validation methods were applied to determine the proper SVMs. The training samples were randomly divided into *k* parts in the training process, each part was used for testing and the others used for training. The obtained MSE each test was recorded and the mean of the MSE acted as the selection criterion for the optimal parameters.

### Comparisons of the forecasting performance

**Figure 3:**
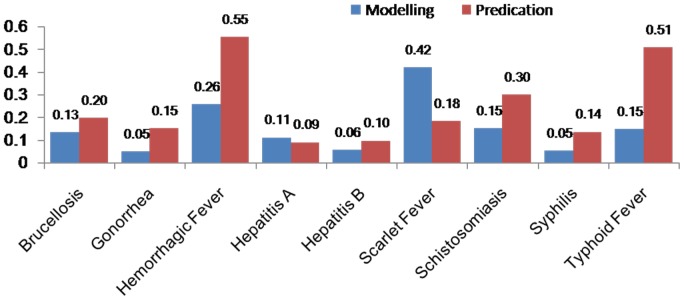
MAPE for Decomposition method (Regression).

**Figure 4:**
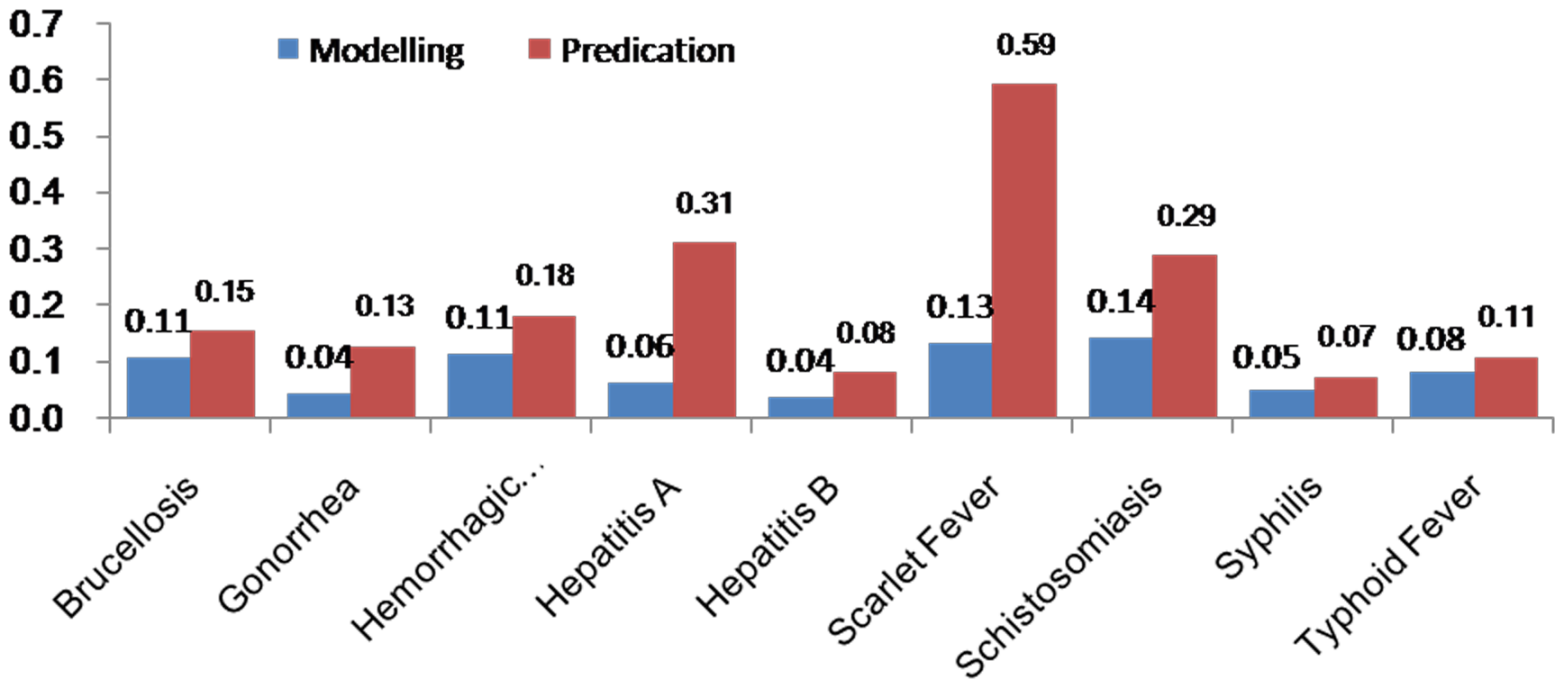
MAPE for Decomposition method (Exponential Smoothing).

**Figure 5:**
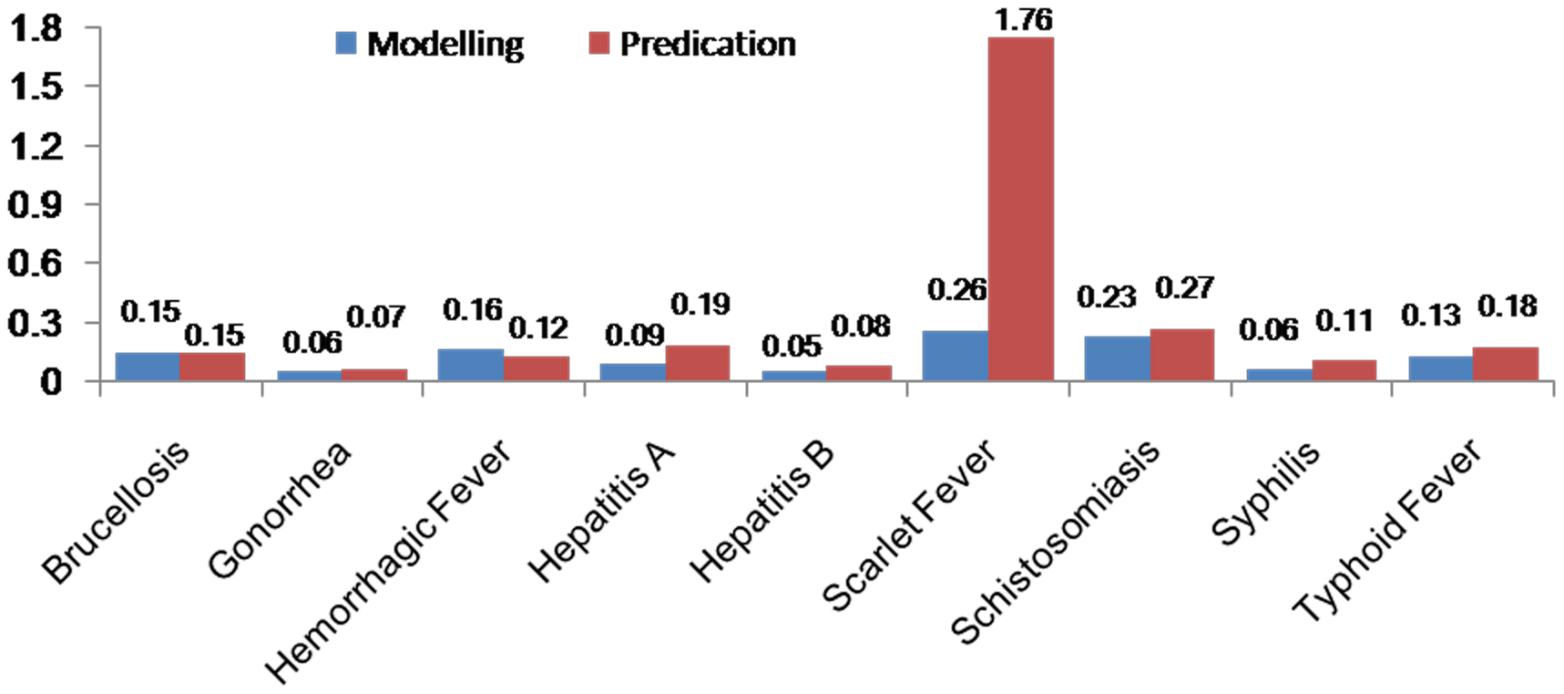
MAPE for ARIMA model.

**Figure 6:**
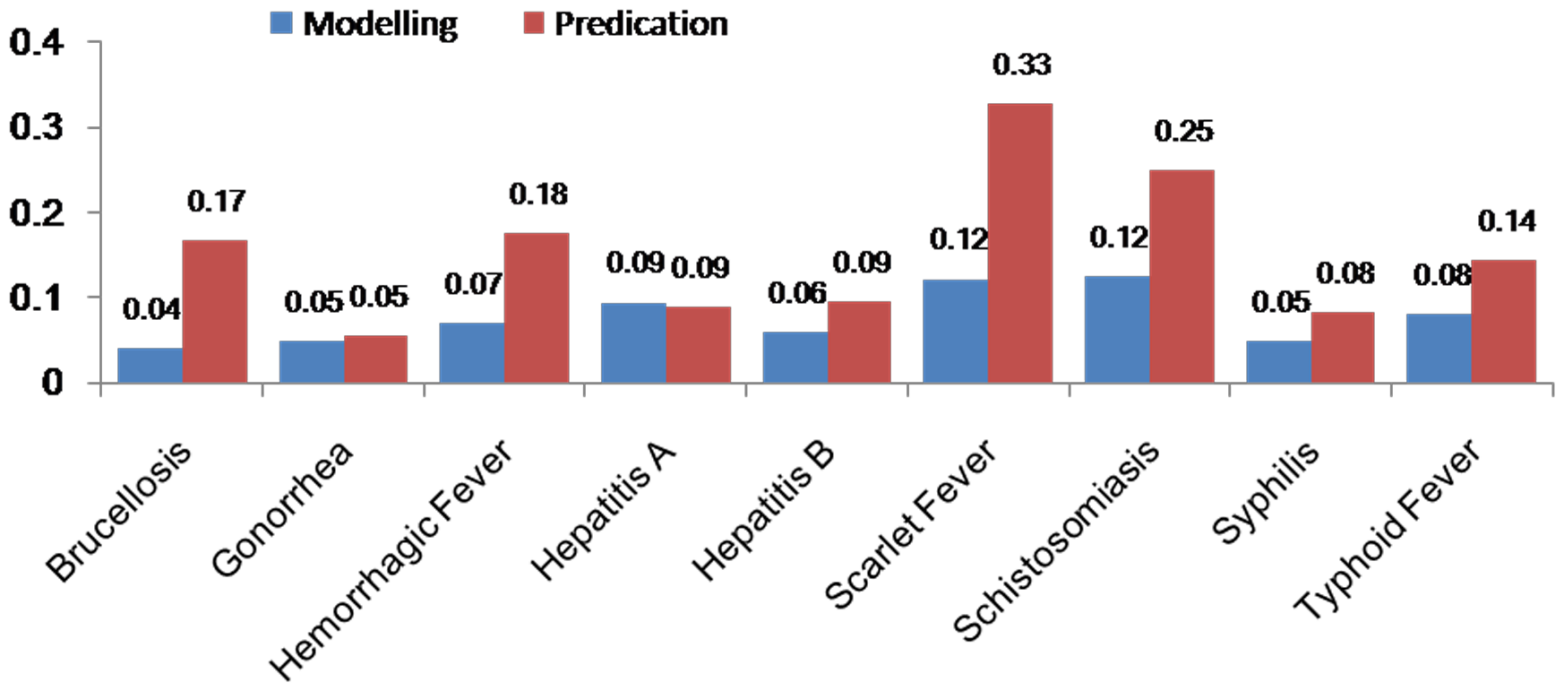
MAPE for SVM model.

**Figure 7:**
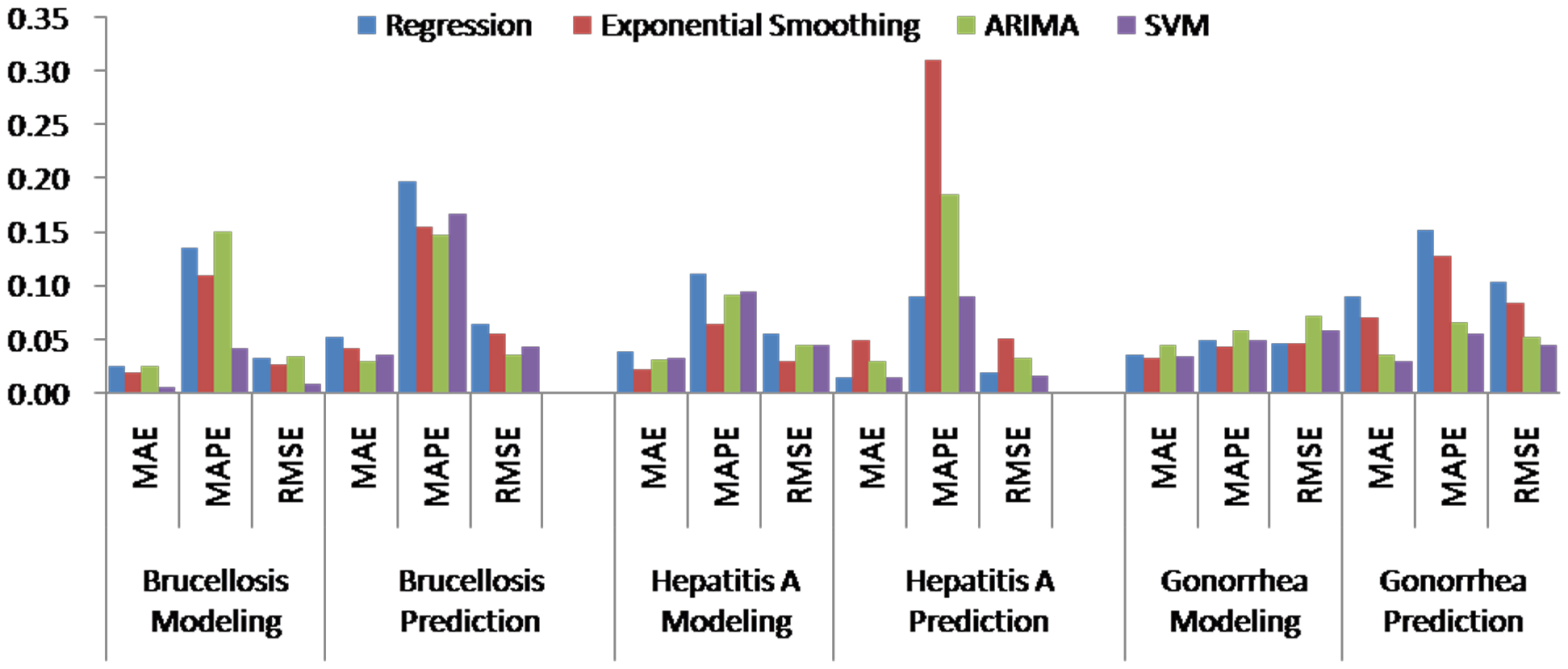
Comparison of the performances of the four different methods (1).

**Figure 8:**
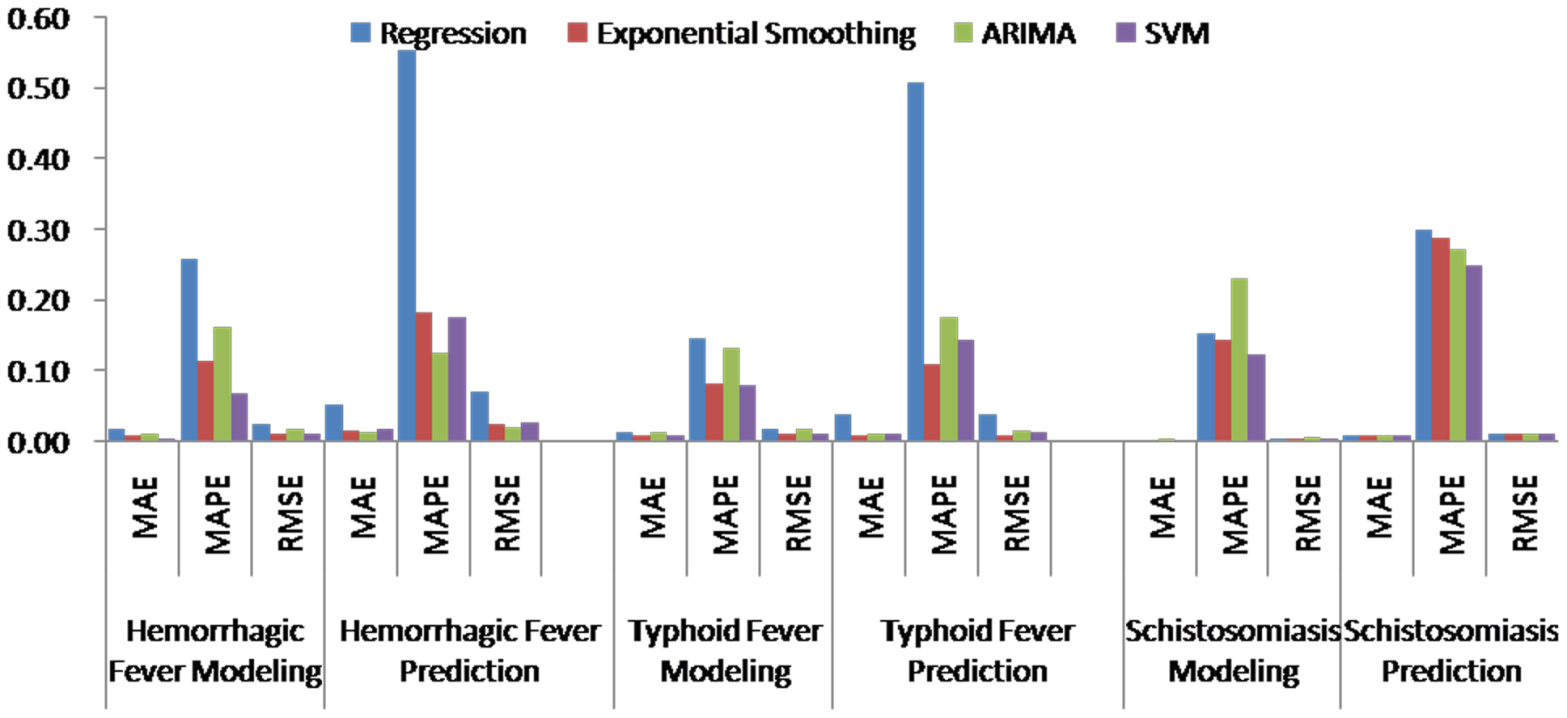
Comparison of the performances of the four different methods (2).

**Figure 9:**
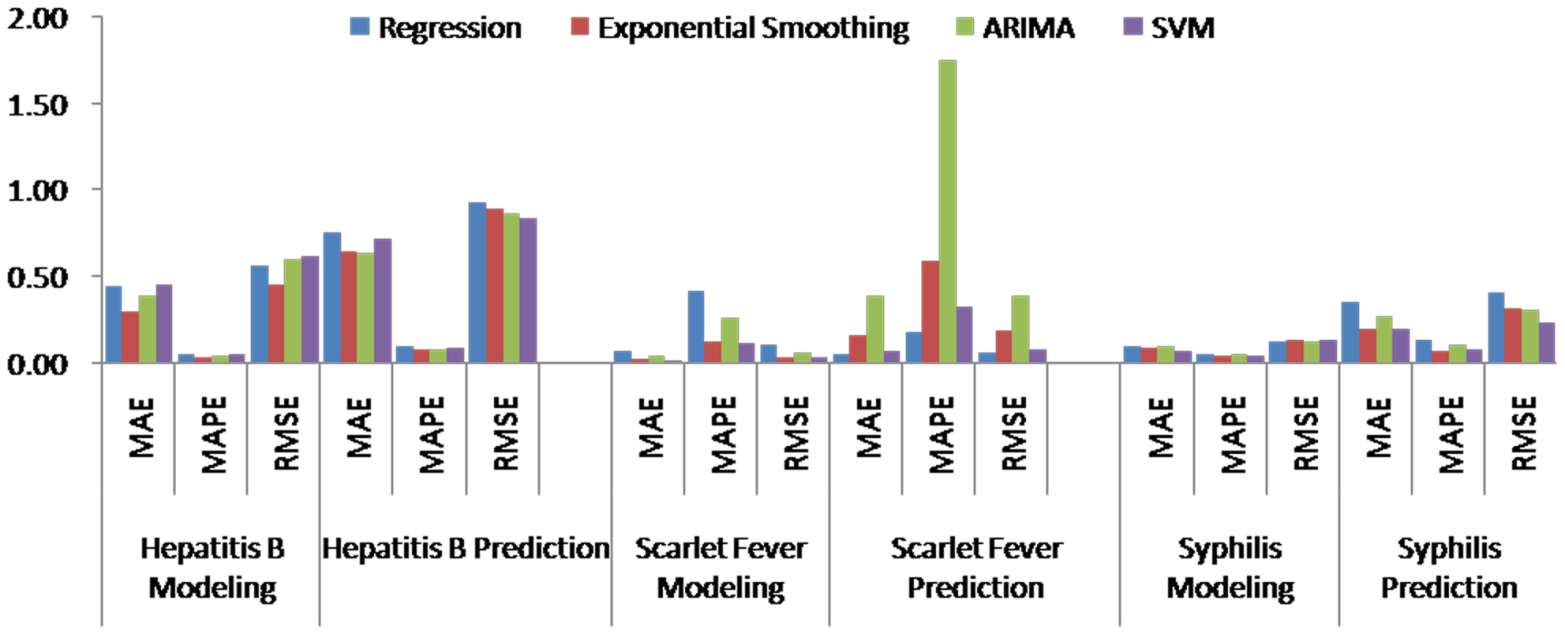
Comparison of the performances of the four different methods (3).

**Figure 10:**
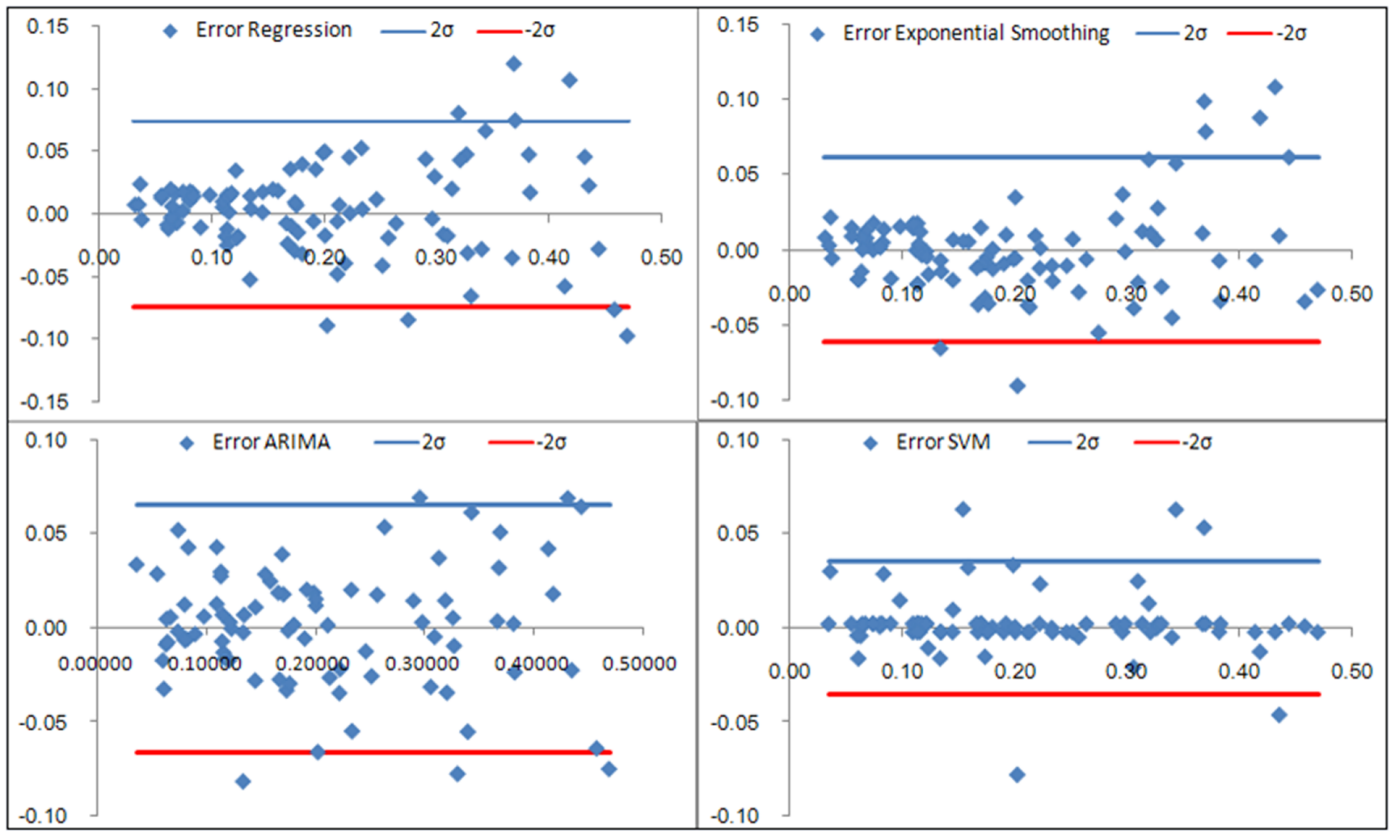
Residual plot of the four methods modeling Brucellosis.

**Figure 11:**
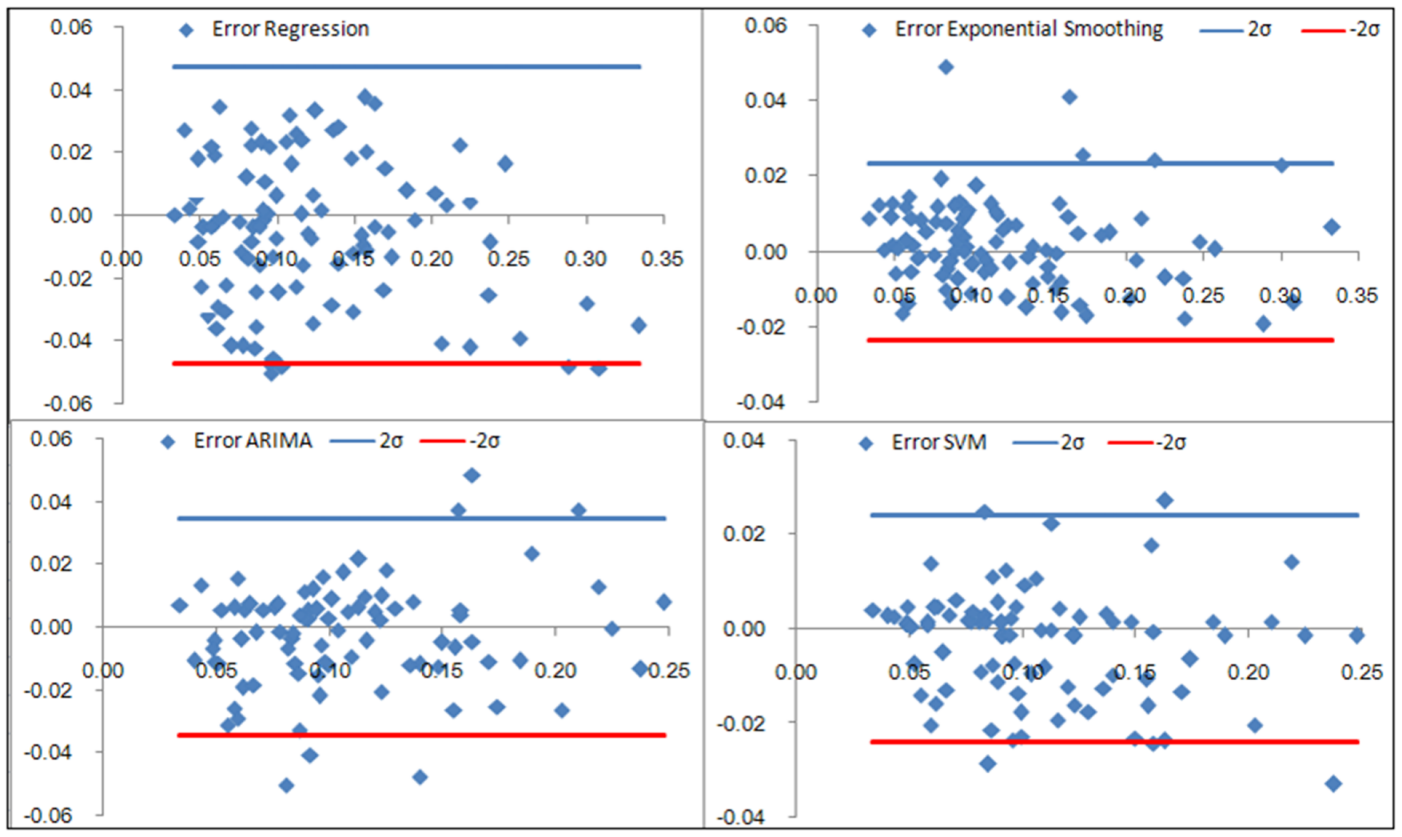
Residual plot of the four methods modeling Typhoid fever.

**Figure 12:**
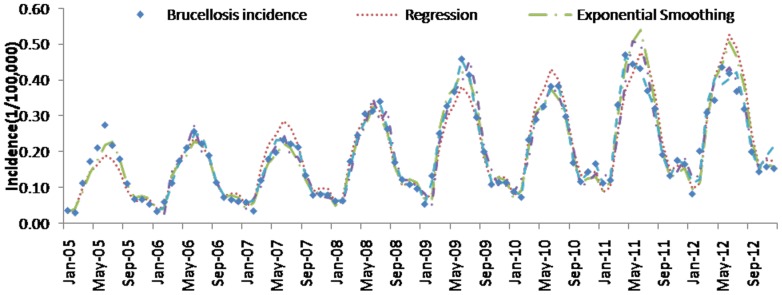
Brucellosis incidence and fitting values predicted by the four methods.

**Figure 13:**
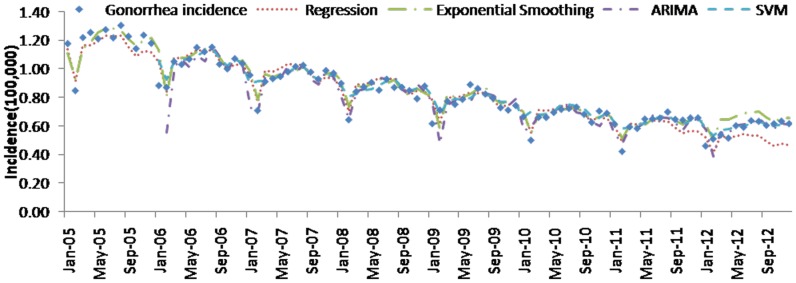
Gonorrhea incidence and fitting values predicted by the four methods.

**Figure 14:**
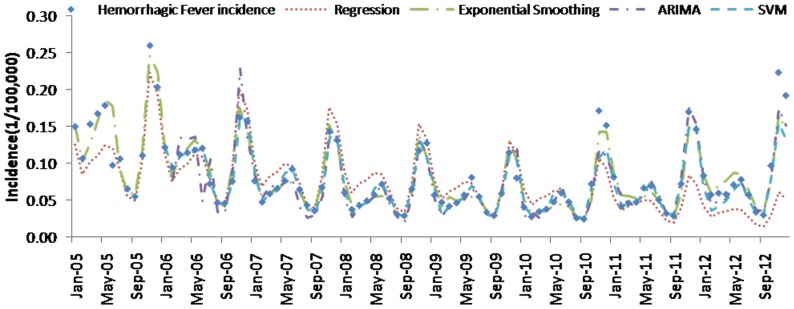
Hemorrhagic fever incidence and fitting values predicted by the four methods.

**Figure 15:**
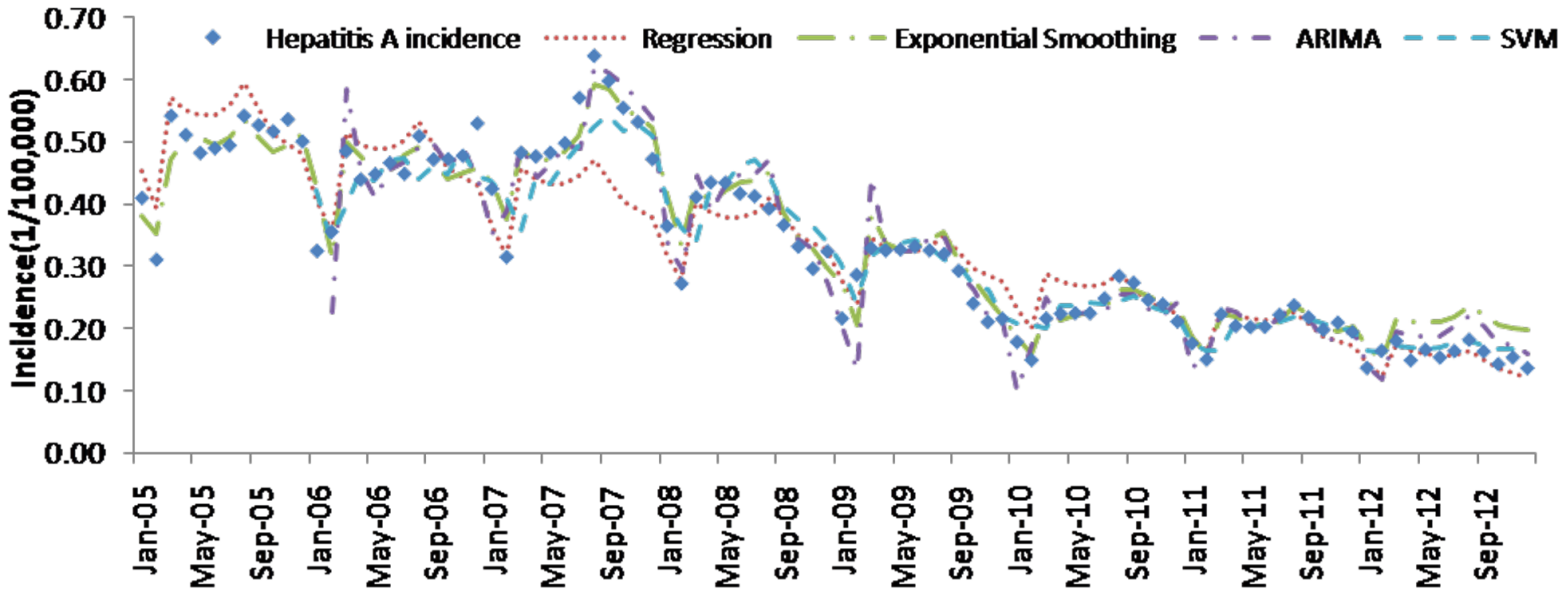
Hepatitis A incidence and fitting values predicted by the four methods.

**Figure 16:**
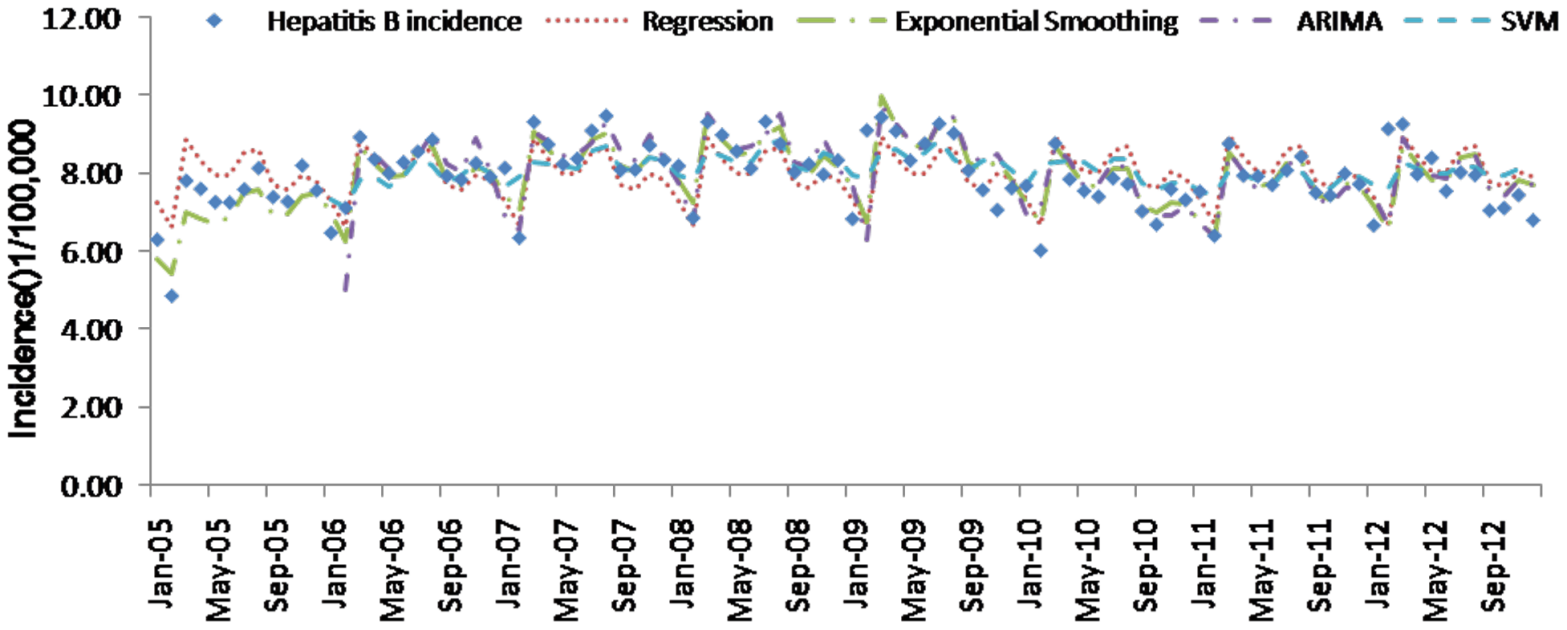
Hepatitis B incidence and fitting values predicted by the four methods.

**Figure 17:**
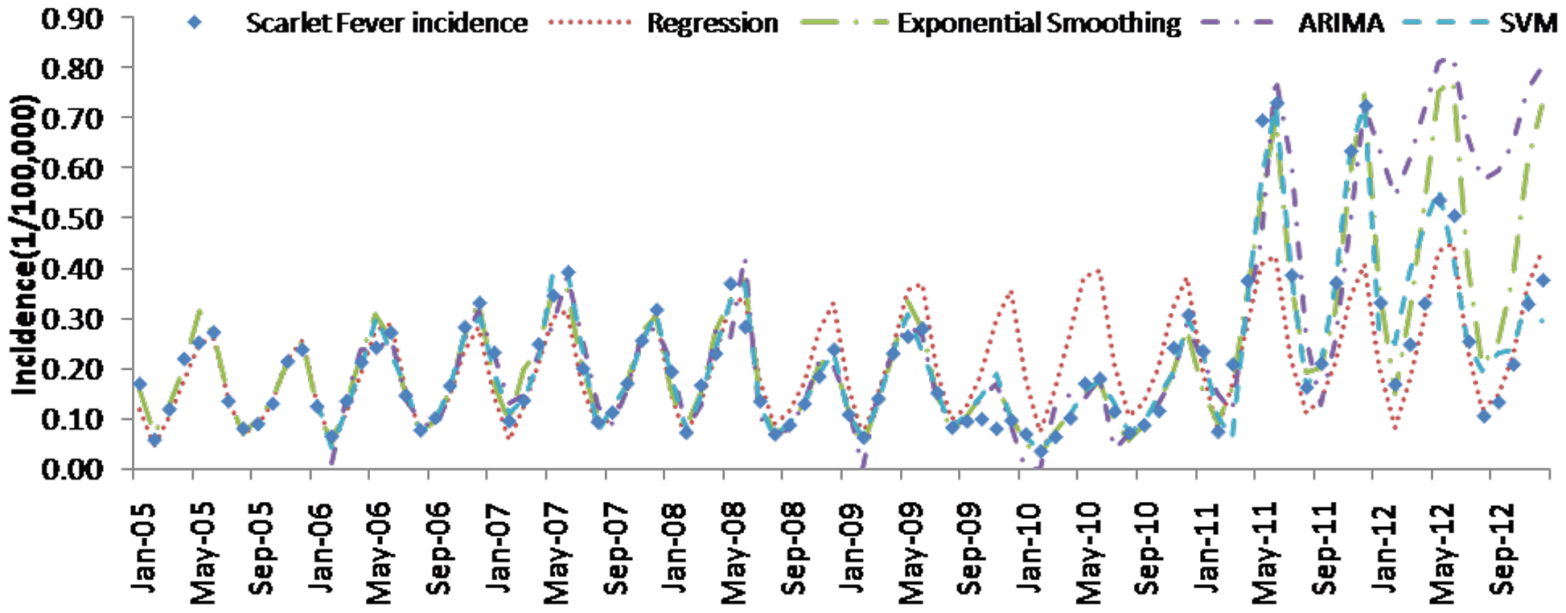
Scarlet fever incidence and fitting values predicted by the four methods.

**Figure 18:**
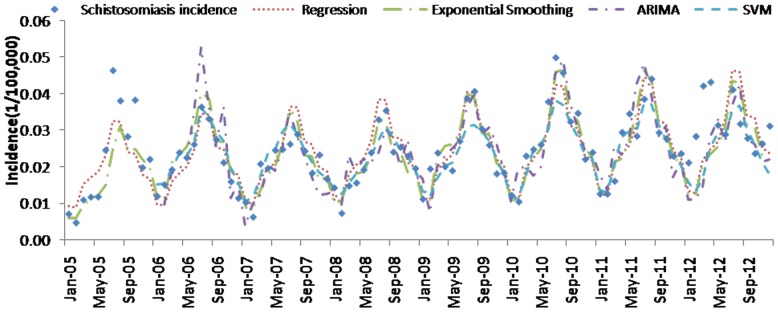
Schistosomiasis incidence and fitting values predicted by the four methods.

**Figure 19:**
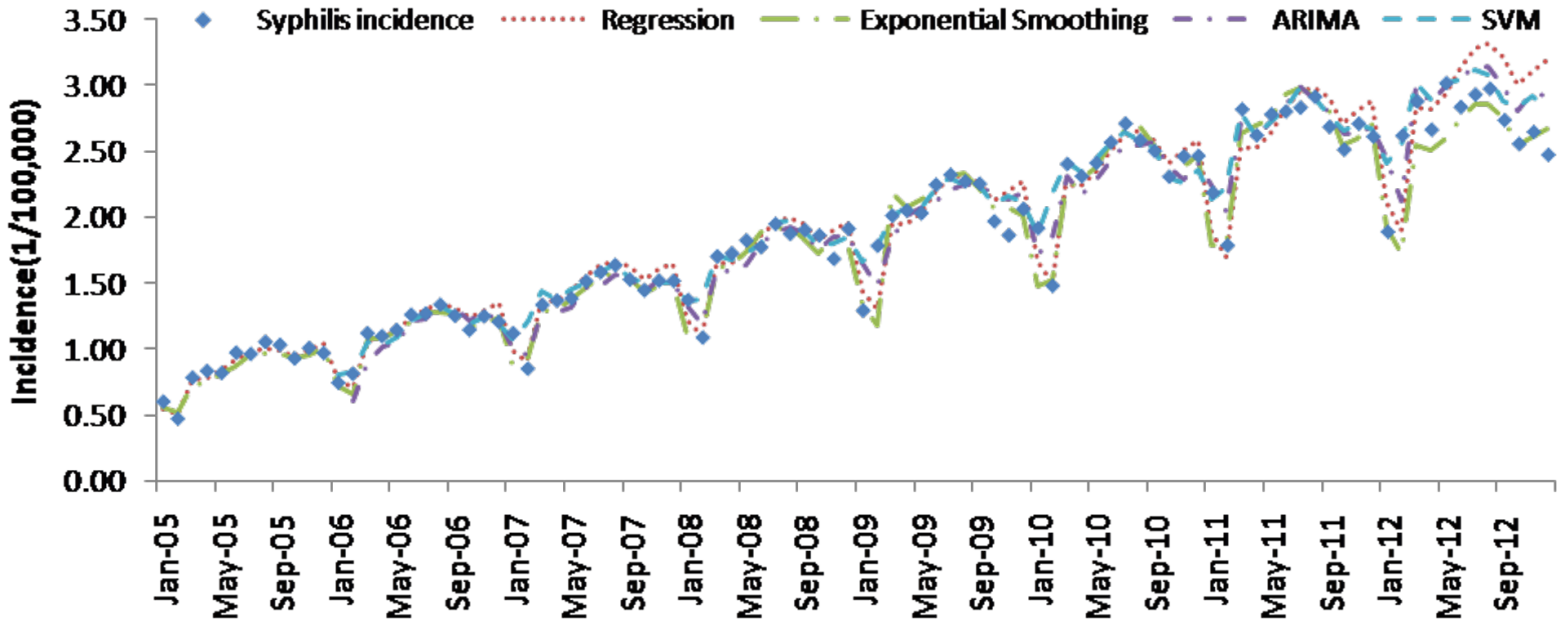
Syphilis incidence and fitting values predicted by the four methods.

**Figure 20:**
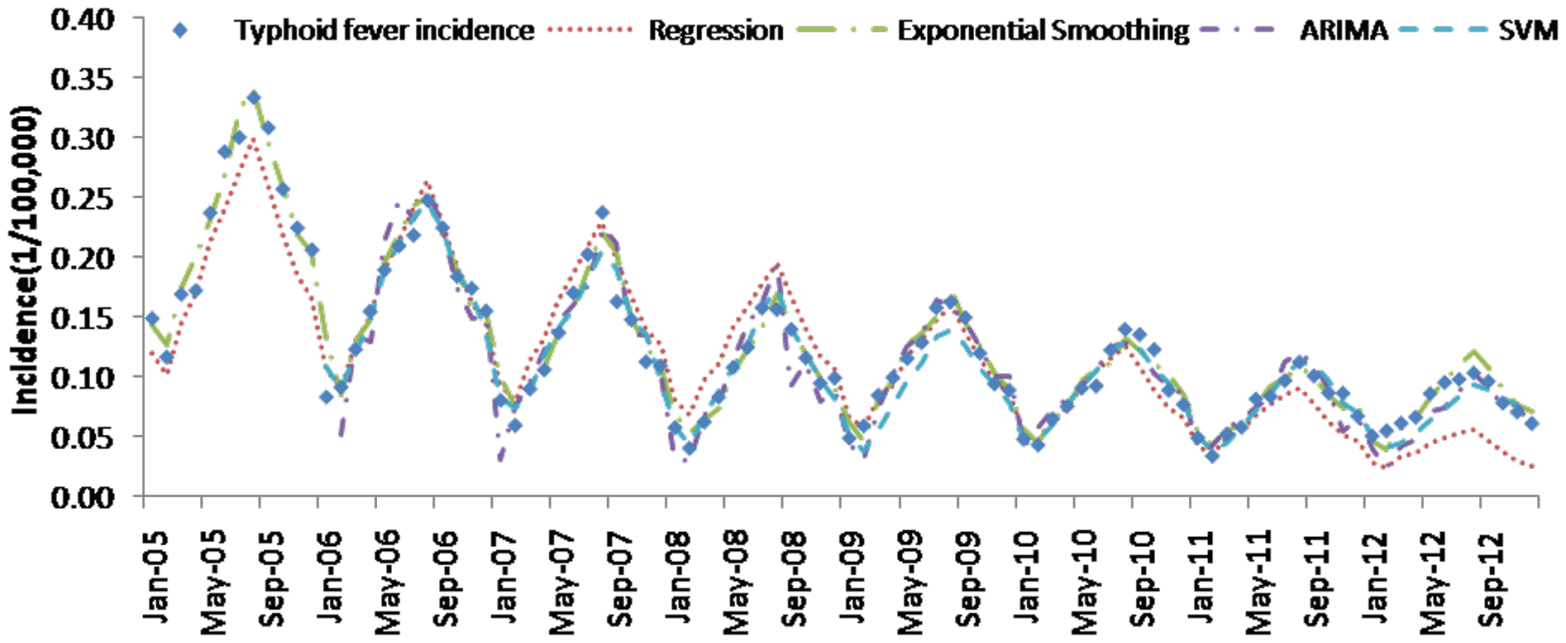
Typhoid fever incidence and fitting values predicted by the four methods.

Table 4 (Comparison of the performance of the three different methods), Figure 3–6(MAPE for ARIMA model) and Figure 7–9 (Comparison of the performances of the three different methods) show the modeling and predication performances of the three methods. Residual plots were made of the four different methods for each disease. The residual plots of Brucellosis and Typhoid fever are presented in this paper as examples (Figure 10–11). The fitting and the forecasting incidences of the four methods for over seven years are graphed in Figure 12–20. Generally, the fitting values and predicated values obtained by all the three methods reasonably matched the real incidence of the infectious diseases. It can also be seen that the performance of the four methods are not quite the same among the different diseases. The standard errors of the MAE, MAPE and MSE are quite small, indicating that these MAE, MAPE and MSE index values are quite stable.

**Table 4:** Comparison of the performance of the four different methods.

Disease	Methods	Modeling						Predication					
		MAE	SE	MAPE	SE	RMSE	SE	MAE	SE	MAPE	SE	RMSE	SE
			(MAE)		(MAPE)		(RMSE)		(MAE)		(MAPE)		(RMSE)
Brucellosis	Regression	0.0240	0.0075	0.1345	0.0873	0.0313	0.0016	0.0520	0.0206	0.1970	0.3565	0.0642	0.0057
	Exponential Smoothing	0.0183	0.0080	0.1084	0.0994	0.0261	0.0018	0.0414	0.0219	0.1546	0.3367	0.0540	0.0060
	ARIMA	0.0247	0.0135	0.1505	0.1210	0.0327	0.0147	0.0285	0.0369	0.1464	0.2396	0.0341	0.0403
	SVM	0.0045	0.0161	0.0402	0.1269	0.0077	0.0033	0.0355	0.0161	0.1667	0.3271	0.0428	0.0051
Gonorrhea	Regression	0.0356	0.0139	0.0481	0.0182	0.0460	0.0056	0.0898	0.0380	0.1510	0.0578	0.1026	0.0190
	Exponential Smoothing	0.0321	0.0139	0.0431	0.0193	0.0457	0.0061	0.0700	0.0401	0.1273	0.0593	0.0835	0.0199
	ARIMA	0.0446	0.0098	0.0570	0.0136	0.0718	0.0128	0.0345	0.0193	0.0659	0.0402	0.0515	0.0238
	SVM	0.0334	0.0397	0.0485	0.0468	0.0570	0.0098	0.0281	0.0547	0.0542	0.0562	0.0436	0.0250
Hemorrhagic Fever	Regression	0.0180	0.0041	0.2580	0.0602	0.0245	0.0004	0.0524	0.0104	0.5528	0.2075	0.0700	0.0014
	Exponential Smoothing	0.0081	0.0043	0.1145	0.0683	0.0110	0.0005	0.0170	0.0105	0.1822	0.2084	0.0240	0.0015
	ARIMA	0.0119	0.0039	0.1628	0.0682	0.0184	0.0050	0.0129	0.0135	0.1246	0.2605	0.0200	0.0188
	SVM	0.0052	0.0049	0.0689	0.0257	0.0105	0.0007	0.0189	0.0148	0.1758	0.1100	0.0285	0.0024
Hepatitis A	Regression	0.0382	0.0332	0.1111	0.0233	0.0539	0.0019	0.0141	0.0071	0.0898	0.1284	0.0176	0.0079
	Exponential Smoothing	0.0209	0.0083	0.0637	0.0378	0.0286	0.0021	0.0482	0.0236	0.3110	0.1283	0.0501	0.0074
	ARIMA	0.0296	0.0101	0.0910	0.0336	0.0435	0.0115	0.0294	0.0090	0.1854	0.0631	0.0319	0.0096
	SVM	0.0313	0.0390	0.0941	0.1278	0.0432	0.0074	0.0132	0.0218	0.0887	0.1352	0.0158	0.0055
Hepatitis B	Regression	0.4468	0.0089	0.0553	0.1670	0.5660	0.1179	0.7544	0.3892	0.0966	0.0261	0.9321	0.0584
	Exponential Smoothing	0.3033	0.0622	0.0384	0.0093	0.4548	0.1309	0.6530	0.1655	0.0817	0.0257	0.8938	0.3839
	ARIMA	0.3922	0.0758	0.0498	0.0099	0.6070	0.0925	0.6425	0.1559	0.0813	0.0186	0.8714	0.1943
	SVM	0.4529	0.2727	0.0583	0.0363	0.6238	0.3054	0.7206	0.1986	0.0942	0.0312	0.8379	0.3997
Scarlet Fever	Regression	0.0718	0.0125	0.4192	0.0785	0.1066	0.0045	0.0514	0.0292	0.1832	0.2896	0.0623	0.0142
	Exponential Smoothing	0.0239	0.0131	0.1321	0.0912	0.0365	0.0049	0.1650	0.0323	0.5909	0.2896	0.1924	0.0151
	ARIMA	0.0416	0.0090	0.2614	0.0738	0.0628	0.0112	0.3888	0.0821	1.7556	0.5018	0.3933	0.0861
	SVM	0.0206	0.0228	0.1214	0.1452	0.0352	0.0061	0.0712	0.0219	0.3278	0.2373	0.0847	0.0110
Schistosomiasis	Regression	0.0032	0.0065	0.1521	0.3112	0.0045	0.0001	0.0095	0.0094	0.2997	0.2923	0.0114	0.0001
	Exponential Smoothing	0.0031	0.0007	0.1440	0.0574	0.0039	0.0000	0.0092	0.0019	0.2882	0.1981	0.0112	0.0000
	ARIMA	0.0048	0.0008	0.2312	0.0499	0.0063	0.0010	0.0088	0.0025	0.2707	0.0692	0.0118	0.0031
	SVM	0.0027	0.0007	0.1242	0.0571	0.0045	0.0000	0.0083	0.0019	0.2490	0.1978	0.0114	0.0000
Syphilis	Regression	0.0987	0.0392	0.0538	0.0398	0.1319	0.0472	0.3557	0.1178	0.1355	0.1446	0.4120	0.1819
	Exponential Smoothing	0.0888	0.0417	0.0501	0.0426	0.1356	0.0544	0.2021	0.1158	0.0741	0.1415	0.3165	0.1762
	ARIMA	0.0999	0.0200	0.0593	0.0126	0.1286	0.0279	0.2722	0.0886	0.1090	0.0370	0.3110	0.1006
	SVM	0.0740	0.0098	0.0477	0.0053	0.1378	0.0029	0.2038	0.1864	0.0828	0.1922	0.2414	0.2675
Typhoid Fever	Regression	0.0145	0.0053	0.1466	0.0508	0.0179	0.0007	0.0389	0.0133	0.5074	0.1687	0.0397	0.0024
	Exponential Smoothing	0.0081	0.0051	0.0813	0.0567	0.0105	0.0007	0.0080	0.0130	0.1086	0.1721	0.0096	0.0024
	ARIMA	0.0133	0.0038	0.1319	0.0429	0.0176	0.0057	0.0121	0.0077	0.1766	0.1070	0.0152	0.0089
	SVM	0.0087	0.0040	0.0797	0.0143	0.0122	0.0010	0.0111	0.0130	0.1435	0.0878	0.0130	0.0032

MAPE is a relative index among the three evaluation indices. We used MAPE to evaluate the general performance for the models to forecast each disease. The MAPEs for each model obtained for each disease in both modeling process and predicating process are shown in Figure 3–6. It was shown that most of the MAPEs obtained by the decomposition (Regression) method in the modeling process are controlled within 30% except scarlet fever (42%). In the predication process, the MAPEs for all infectious disease are controlled within 30% except hemorrhagic fever (55%), and typhoid fever (51%). The decomposition (Regression) methods had bad performance in fitting scarlet fever incidence and predicating those of hemorrhagic fever and typhoid fever. All of the MAPEs obtained by decomposition (Exponential Smoothing) method in the modeling process were controlled within 15%. The method generally had a good fit in the modeling process. In the predication process, the MAPEs for all infectious diseases were controlled within 30% except scarlet fever (59%) and Hepatitis A(31%). The decomposition (Exponential Smoothing) methods had bad performance in predicating scarlet fever incidence. The MAPEs obtained by ARIMA model in the modeling process method were controlled within 30%. In the predication process, the MAPEs for the 9 kinds of infectious diseases were controlled within 30% except scarlet fever (175%). The ARIMA model had good performance in the fitting process of all the infectious diseases selected. But it had bad performance in forecasting scarlet fever. The MAPEs obtained by SVM model in the modeling process are controlled within 15%. In the predication process, the MAPEs for the 9 kinds of infectious diseases were controlled within 20% except scarlet fever (33%) and Schistosomiasis (25%). The SVM based model had good performance in the fitting process and predicting process of all the infectious diseases selected.

To compare the performance the different models for different diseases, different evaluation indices were emphasized. MAPE is emphasized for lower level incidence disease (annual mean incidence <0.1/100,000) such as Schistosomiasis (0.0245/100,000) and Hemorrhagic Fever (0.0814/100,000). RMSE is emphasized for higher level incidence disease (mean incidence >1/100,000), such as Hepatitis B (7.9335/100,000) and syphilis (1.8461/100,000). MAE was emphasized for medium level incidence disease (0.1/100,000<mean incidence <1/100,000) including Hepatitis A (0.3356/100,000), gonorrhea (0.8329/100,000), scarlet fever (0.2131/100,000), typhoid fever (0.1242/100,000) and brucellosis (0.1975/100,000). The performances of the three methods for gonorrhea, hepatitis B, Schistosomiasis and Syphilis ranked in descending order were: SVM, ARIMA, exponential smoothing and regression. The performances of the three methods for Hepatitis A ranked in descending order were: SVM, regression, exponential smoothing and ARIMA. The performances of the three methods for Brucellosis and Hemorrhagic fever ranked in descending order were: ARIMA, SVM, exponential smoothing and regression. The performances of the four models for Scarlet Fever ranked in descending order were: regression, SVM, exponential smoothing and ARIMA. The performances of the four models for typhoid fever ranked in descending order were: exponential smoothing, ARIMA, SVM and regression. SVMs performed best in forecasting gonorrhea, hepatitis A, hepatitis B, Schistosomiasis and Syphilis. ARIMA performed best in forecasting Brucellosis and Hemorrhagic Fever and performed the worst in forecasting Scarlet Fever. Exponential smoothing performed best in forecasting typhoid fever, but worst in hepatitis A. Regression method performed best in forecasting scarlet fever, however the worst in Brucellosis, Gonorrhea, Hemorrhagic Fever, Schistosomiasis, Syphilis and typhoid fever. The exponential smoothing method performs better than regression decomposition method except in the case of hepatitis A and scarlet fever.

## Discussion

The early recognition of epidemic behavior is significantly important for epidemic disease control and prevention. The effectiveness of statistical models in forecasting future epidemic disease incidence has been proved useful [37]. The surveillance system is a good way to collect and analyze infectious disease data. With high quality surveillance data, the epidemic behavior may be accurately detected and forecasted. Discussion of the forecasting techniques is very important. In the present study, we conducted a comparative study of four typical time series investigations in the forecasting of the epidemic pattern of nine types of infectious diseases, namely two decomposition methods (regression and exponential smoothing), ARIMA model, and SVMs based model. We have also compared the differences among these methods in both principle and practical aspects.

In principle, the decomposition method can break down the original into different parts. The seasonal factor can be expressed in the form of seasonal indices. The series after seasonal pattern removal can be modeled with regression methods or exponential smoothing, etc. Time series decomposition models do not involve a lot of mathematics or statistics; they are relatively easy to explain to the end user. The ARIMA model can grasp the historical information by (1) AR to consider the past values, and (2) MA to consider the current and previous residual series. The ARIMA model is popular because of its known statistical properties and the well-known Box–Jenkins methodology in the modeling process. It is one of the most effective linear models for seasonal time series forecasting. In contrast, the SVMs time series models capture the historical information by nonlinear functions. With flexible nonlinear function mapping capability, support vector machine can approximate any continuous measurable function with arbitrarily desired accuracy.

In practical matters, the building of the decomposition methods generally involves two parts: (1) extraction of the seasonal indices to express the seasonal pattern hidden in the infectious disease time series, and (2) regression methods to model the long trend pattern. The building of the ARIMA model requires the determination of differencing orders (*d*, *D*), and operators (*p*, *q*, *P*, *Q*), as well as the estimation of model parameters in the autoregressive and moving average polynomials. The construction of SVMs requires the determination of three parameters, namely, 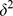, C, 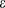. The time series data should be transformed into the input matrix and the output matrix, and then be put into the support vector machine. Certain training accuracy goals should be assigned before training.

Based on the three forecasting measured errors (MAE, MAPE, MSE), and the visualization of the forecasted values, the empirical evidence is that no one method completely dominated the others. However, the present study shows that support vector machine generally outperforms the conventional ARIMA model and decomposition methods. The ARIMA model has been proved an effective linear model to effectively capture a linear trend of the infectious disease series. The decomposition methods generally perform better when the series conform to the decomposition hypothesis. The linear regression hypothesis seems to be more rigid on the season moved series than exponential smoothing.

The advantage of decomposition is that decomposition models do not involve a lot of mathematics or statistics; they are relatively easy to explain to the end user. This is a major advantage because if the end user has an appreciation of how the forecast was developed, he or she may have more confidence in its use for decision making. The disadvantage of decomposition methods is that the hypothesis may be too strong for the epidemic behavior, so that the model may not perform well sometimes. The ARIMA model has advantages in its well-known statistical properties and effective modeling process. It can be easily realized through mainstream statistical software. The model can be used when the seasonal time series are stationary and have no missing data. The disadvantage of the ARIMA model is that it can only extract linear relationships within the time series data. it may not work well for the occurrence of an infectious disease which can be affected by various factors, including many meteorological and various social factors, namely, the occurrence of the disease does not necessarily associate with the historical data in linear relationship. Our study suggested that nonlinear relationships may exist among the monthly incidences of many diseases such as scarlet fever, so that the ARIMA model did not efficiently extract the full relationship hidden in the historical data. Support vector machines are potentially useful endemic time series forecasting methods because of their strong nonlinear mapping ability and tolerance to complexity in forecasting data. SVMs have very good learning ability in time series modeling. SVMs have unique advantages compared with other machine learning methods, such as neural networks. For example, the SVMs implement the structural risk minimization principle, which leads to better generalization than neural networks that implement the empirical risk minimization principle. SVMs also have fewer free parameters than neural networks [38].

What is more, the scarlet fever incidence shown in figure 17 (Scarlet fever incidence and fitting values predicted by the four methods) indicated that the average incidence from 2011 to 2012 was higher than that in the previous six years (2005–2010). The phenomenon that the incidence level changed greatly through time was called level shift by Tsay, R. S. in 1988.[39] Since the ARIMA model is in fact a regression of the present incidence value on the past values and residuals, it is of high risk that level shift would likely affect the forecasting performance of ARIMA model. Therefore, statisticians and time series analysts have tried to overcome the effect of level shift for many years. In our paper, it is interesting that, as presented in Table 4, the MAE, MAPE, RMSE and their standard errors of ARIMA model are larger than those of decomposition model, SVM and exponential smoothing method. This result in our paper suggests that the other three methods may serve as a better way than SARIMA model in analyzing time series in the presence of level shift.

The limitations of the study should also be acknowledged. First, only eight-years of incidence data were obtained because the Chinese National Surveillance System for Infectious Disease was established only in 2004. The relatively short length of the series may influence the forecasting efficacy of the different methods. Second, we only predicted the infectious disease incidence with the four typical forecasting methods. The findings based on a specific disease may not be repeatable when used on other cases. What is more, there are some other hypotheses on the long term trend in decomposition methods, such as generalized models which assume a nonlinear function among the time series. Many other models were developed to make up deficiencies of ARIMA, such as GARCH, etc. SVM is only one of the typical machine learning techniques. In this paper, we only choose four very typically used time series methods to make a comparison.

Infectious diseases pose a significant threat to human health. The establishment of epidemiological surveillance system greatly facilitates the implement of strategic health planning, such as vaccination costs and stocks. More research on the accurate prediction of the epidemiological events based on surveillance data should be conducted, and more sophisticated forecasting techniques should be applied and compared in practice.
