# Correction: Applications and Comparisons of Four Time Series Models in Epidemiological Surveillance Data

**DOI:** 10.1371/journal.pone.0091629

**Published:** 2014-02-28

**Authors:** The PLOS ONE Staff

The name of a funding organization in the Funding statement is incorrect. The Funding statement should read: “The research is funded by National science and technology major special project "Data mining and analysis of the surveillance data of five syndrome pathogen (grant no. 2012ZX10004201-006)." The funders had no role in study design, data collection and analysis, decision to publish, or preparation of the manuscript.”

## References

[1] Zhang X, Zhang T, Young AA, Li X (2014) Applications and Comparisons of Four Time Series Models in Epidemiological Surveillance Data. PLoS ONE 9(2): e88075 doi:10.1371/journal.pone.0088075 24505382 10.1371/journal.pone.0088075 PMC3914930

